# Bioengineering *in vitro* models of embryonic development

**DOI:** 10.1016/j.stemcr.2021.04.005

**Published:** 2021-05-11

**Authors:** Ananya Gupta, Matthias P. Lutolf, Alex J. Hughes, Katharina F. Sonnen

**Affiliations:** 1 Department of Bioengineering, School of Engineering and Applied Science, University of Pennsylvania, Philadelphia, PA 19104, USA; 2 Laboratory of Stem Cell Bioengineering, Institute of Bioengineering, School of Life Sciences and School of Engineering, École Polytechnique Fédérale de Lausanne (EPFL), Lausanne, 1015 Vaud, Switzerland; 3 Institute of Chemical Sciences and Engineering, School of Basic Science, École Polytechnique Fédérale de Lausanne (EPFL), Lausanne, 1015 Vaud, Switzerland; 4 Department of Cell and Developmental Biology, Perelman School of Medicine, University of Pennsylvania, Philadelphia, PA 19104, USA; 5 Hubrecht Institute-KNAW (Royal Netherlands Academy of Arts and Sciences) and University Medical Center Utrecht, Utrecht, the Netherlands

**Keywords:** embryonic development, *in vitro* models, bioengineering, stem cells, microfluidics, 3D matrix, optogenetics, blastoids, gastruloids, organoids

## Abstract

Stem cell-based *in vitro* models of embryonic development have been established over the last decade. Such model systems recapitulate aspects of gametogenesis, early embryonic development, or organogenesis. They enable experimental approaches that have not been possible previously and have the potential to greatly reduce the number of animals required for research. However, each model system has its own limitations, with certain aspects, such as morphogenesis and spatiotemporal control of cell fate decisions, diverging from the *in vivo* counterpart. Targeted bioengineering approaches to provide defined instructive external signals or to modulate internal cellular signals could overcome some of these limitations. Here, we present the latest technical developments and discuss how bioengineering can further advance the optimization and external control of stem cell-based embryo-like structures (ELSs). *In vitro* models combined with sophisticated bioengineering tools will enable an even more in-depth analysis of embryonic development in the future.

## Teaser

In this review, Lutolf and coworkers discuss how bioengineering approaches can further advance the optimization and external control of stem cell-based embryo-like structures. *In vitro* models of embryonic development combined with sophisticated bioengineering tools will enable an even more in-depth analysis of embryonic development in the future.

## Introduction

How multicellular organisms develop from single cells has fascinated scientists for centuries. Ordered intracellular events, intercellular communication, and interactions with the environment lead to emergent self-organization of the embryo and ultimately an adult organism. How this self-organization proceeds and which mechanisms determine the reproducibility and robustness of development are still key questions to date.

**Figure 1:**
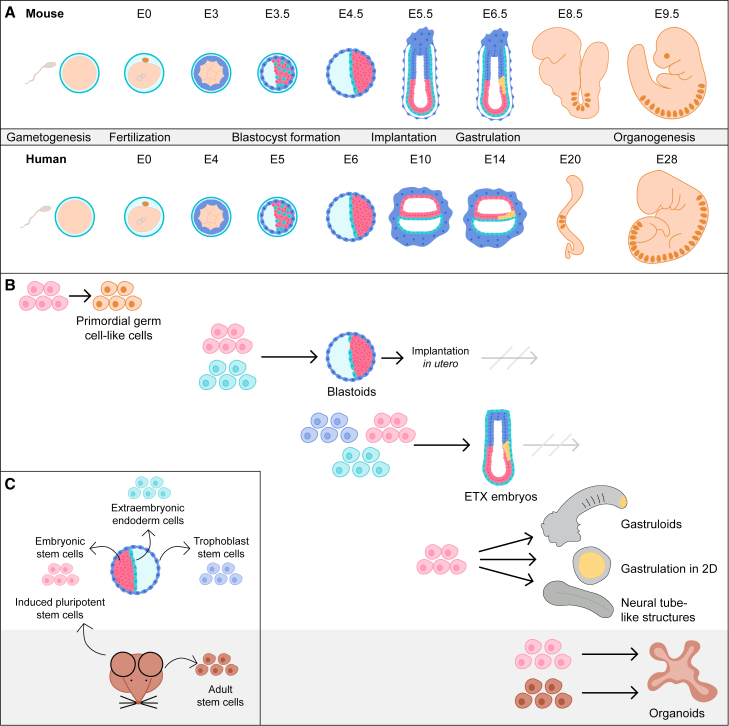
Stem cell-based *in vitro* models of embryonic development (A) Schematic representation of mouse and human embryonic development including developmental time in embryonic days and key developmental processes. (B) Schemes of *in vitro* models of embryonic development and cell types required for their generation are shown. (C) Derivation of stem cells for the generation of *in vitro* models. ESCs are derived from the ICM, XEN cells from the PE, and TSCs from the trophectoderm of blastocysts. Adult tissue-specific stem cells are derived from the corresponding adult tissue. Adult cells can also be reprogrammed to iPSCs, similar in pluripotency state to ESCs.

*In vitro* culture of mammalian embryos has enabled the study of embryonic development. However, embryos are often only available in low numbers, hindering high-content, high-throughput experimental approaches, such as those based on modern omics techniques. Moreover, for ethical reasons, the *in vitro* culture of human embryos is so far restricted by the “14-day rule” to the pre-gastrulation stage. To address these limitations, *in vitro* models of embryonic development and organogenesis have been developed that build on the self-organizing capacity of pluripotent and adult stem cells (Figure 1). While such cultures have been used successfully to study aspects of development, optimized protocols are regularly published that allow the formation of embryo-like structures (ELSs) with ever closer resemblance to *in vivo* embryos at ever later stages. Similarly, *in vitro* models of organogenesis recapitulate aspects of organ formation, but are still incomplete in their morphogenesis and the presence and spatial organization of the required tissues.

In this perspective, we discuss opportunities for *in vitro* models across different stages of embryonic development. We highlight how bioengineering approaches might overcome current limitations and how they can contribute to guiding or optimizing self-organization of *in vitro* models. General principles include setting initial and boundary conditions that guide development and determine sites of organ formation *in vitro*. This can, for example, be achieved through direct construction approaches such as bioprinting to approximate the scale and spatial heterogeneity of developing systems. However, this will likely never achieve sufficient spatial resolution to generate accurate tissue simulacra. Alternatively, tissue construction can be guided across scales by replicating patterning programs including morphogen signaling or mechanical forces to induce shape changes.

Bringing together developmental biology and bioengineering perspectives could make ELS a viable substitute for mammalian embryos in scientific research.

## Germ cell engineering

**Figure 2:**
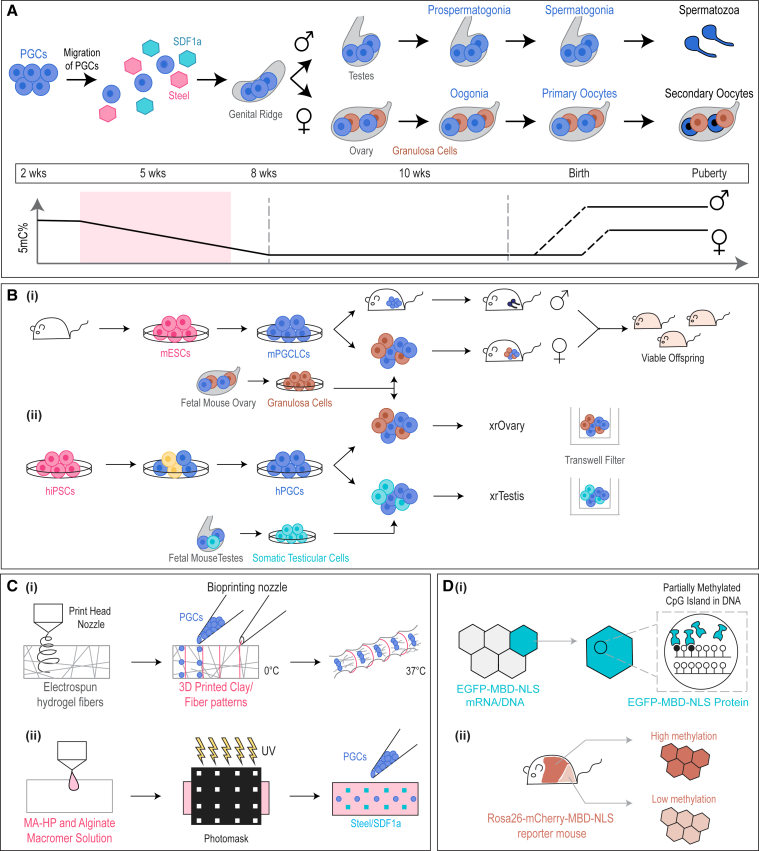
Bioengineering tools to model human germ cell development (A) Schematic representation of human germ cell development including developmental milestones, gestational timeline, and levels of genome methylation. (B) Germ cell lineage-specific differentiation schemes for mESCs and hiPSCs: (i) mPGCLCs transplanted into neonatal mouse testis depleted of endogenous germ cells give rise to healthy sperm. mPGCLCs aggregated with granulosa cells induce formation of oocytes capable of giving rise to viable offspring when fertilized with mPGCLC-derived sperm. (ii) hiPSC-derived PGCs aggregated with granulosa cells from mouse ovary or with somatic cells from mouse testes are cultured at air-liquid interface, termed xenogenic reconstituted ovary (xrOvary) or xenogenic reconstituted testis (xrTestis), respectively. (C) (i) hiPSCs-derived PGCs may be encapsulated into an electrospun thermoresponsive hydrogel. Following crosslinking, patterns are 3D printed. Upon exposure to 37°C, the hydrogel undergoes non-uniform swelling to induce passive migration in PGCs. (ii) Methacrylated heparin (MA-HP) and alginate macromer solution may be used to pattern Steel/SDF1a factors in order to facilitate active migration of encapsulated PGCs. (D) (i) Green fluorescent methylation binding proteins (EGFP-MBD-NLS) can be used for evaluating methylation dynamics in embryos. (ii) The methylation reporter mouse Methyl*RO* (Rosa26-mCherry-MBD-NLS) is used for live imaging of methylation dynamics.

Infertility caused by defects in gametogenesis is staggeringly common in humans. This has motivated the development of new bioengineering tools to mimic the embryonic context of germ cell differentiation *in vitro*. Human germ cells are specified from primordial germ cells (PGCs), the precursors to oogonia and pro-spermatogonia, which then undergo meiosis to form ova and spermatozoa. PGC induction is governed by signals from extraembryonic tissues prior to gonad development. PGCs are initially found in the dorsal wall of the yolk sac. Subsequently, guided by chemokines, they migrate through the developing hindgut and dorsal mesentery and colonize the genital ridge (precursors to ovaries or testes). PGCs then undergo epigenetic reprogramming and sex-specific differentiation (Kobayashi et al., 2017) (Figure 2A).

Much of the knowledge about the specification of the germ cell lineage has been derived from *in vitro* studies. For instance, mouse embryonic stem cells (mESCs) have been differentiated into mouse primordial germ cell-like cells (mPGCLCs) (Hayashi et al., 2011). To finish gametogenesis, mPGCLCs have been transplanted into neonatal mouse testes depleted of endogenous germ cells. Similarly, mPGCLCs can be aggregated with embryonic ovarian somatic cells to induce the formation of oocytes. The resulting spermatozoa and ova can undergo fertilization and give rise to healthy offspring (Hayashi et al., 2012) (Figure 2B). Furthermore, identification of transcription factors that control mouse oocyte growth has enabled engineering of directly induced oocyte-like cells from pluripotent stem cells (PSCs) (Hamazaki et al., 2021).

Although mouse models have provided useful insights into the process of germ cell development, there are critical differences between mouse and human embryonic germ cell development, including timescale, demethylation patterns, and transcriptional regulation (Murase et al., 2020; Sugawa et al., 2015). In parallel, human induced PSCs (hiPSCs) have been differentiated into primordial germ cell-like cells (hPGCLCs) *in vitro* (Irie et al., 2015; Sasaki et al., 2015; Sugawa et al., 2015). These hPGCLCs can be cultured at the gas-liquid interface of a transwell culture system in aggregates with mouse testicular or ovarian somatic cells as xenogenic reconstituted testis (xrTestis) or ovary (xrOvary). Within 5 months, these hPGCLC-derived cells undergo genome-wide demethylation to form pro-spermatogonia or oogonia-like cells (Hwang et al., 2020; Yamashiro et al., 2018) (Figure 2B). In addition to studying the biology of human germ cell development, these results offer the possibility of generating hiPSC-derived genome-matched germ cells as candidates to treat infertility (Zhao et al., 2020).

Despite recent advances in germ cell engineering, processes such as the migration of PGCs have not been modeled *in vitro*. The integration of PGCs in artificial shape-morphing hydrogels could replicate the tissue expansion experienced by PGCs as they migrate (Figure 2C). Hydrogels can be made to undergo complex shape transformations through non-uniform swelling (Chen et al., 2018). Recapitulating the migration of PGCs through a dynamic microenvironment may be further enhanced by the use of a three-dimensional (3D) hydrogel photopatterned with chemokines (Figure 2C). For instance, the development of a heparin-micropatterned dual crosslinked alginate hydrogel allows for location-specific immobilization and controlled, sustained release of chemokines for regulation of encapsulated stem cell behavior (Jeon et al., 2018). Such *in vitro* models would also complement live imaging for epigenetic changes through genetically encoded DNA methylation sensors during the migration process (Ingouff et al., 2017; Ueda et al., 2014) (Figure 2D).

### Engineering pre-implantation development

The first steps of embryonic development culminate in the formation of a blastocyst. Blastocysts consist of an outer cell layer of trophectoderm (TE) surrounding the inner cell mass (ICM), which forms the epiblast (Epi) and primitive endoderm (PE). Epi cells give rise to the embryo proper, while PE and TE form extraembryonic endoderm and trophoblast cells, respectively. This stage has been modeled *in vitro* by aggregating embryonic stem cells (ESCs) and trophoblast stem cells (TSCs), which induces the self-organization of blastocyst-like cultures, termed blastoids, recapitulating aspects of cell specification, spatial organization and implantation ability (Figure 1B) (Rivron et al., 2018b). Recently, the first protocols to generate human blastoids have been published (Liu et al., 2021; Yu et al., 2021), which is an exciting prospect for the study of human pre-implantation development.

For efficient blastoid formation, the number of input cells is critical. Microwells have been used to allow the aggregation of specific cell numbers (Li et al., 2019; Rivron et al., 2018b; Sozen et al., 2019). Together with advanced cell-handling robots they could ensure precise input cell numbers per culture well. Microfluidic chips with defined dimensions have also been used to control the size of growing human ESC aggregates within a channel of extracellular matrix (ECM) (Zheng et al., 2019). Such microfluidic flow chambers and robotic setups may be adapted to allow the stepwise assembly of ELS by consecutively adding the required cell types and growth factors. This would allow these models to be transferred to larger assay pipelines such as those used for drug development (e.g., toxicology assessment).

To promote the formation of an internal layer of PE cells within mouse blastoids, a cocktail of signaling modulators has been described (Vrij et al., 2019). Another approach is the use of extended PSCs (EPS cells) instead of ESCs for blastoid generation (Li et al., 2019; Sozen et al., 2019). Blastoids implant and begin early stages of post-implantation development *in utero*, but morphogenesis does not progress and blastoids are soon resorbed (Li et al., 2019; Rivron et al., 2018b; Sozen et al., 2019). One important step toward improving later development is the efficient formation of all required cell lineages at correct locations with proper gene expression patterns (Posfai et al., 2021). Bioengineering approaches might help to externally modulate regulatory processes to build blastoids that are able to develop further upon implantation.

Mechanical properties of cells and morphogenetic changes are known to affect cell fate specifications within the morula. These processes result in the generation of outer TE and ICM. It has been shown that heterogeneous contractility of cells controls cell sorting into TE and ICM (Maître et al., 2016). In addition, the liquid-filled lumen of the blastocyst exerts mechanical force on cells and provides growth factors to control development (Chan et al., 2019; Dumortier et al., 2019). *In vitro*, we could build on such findings to guide development of ELS. Contractility can for instance be perturbed by small molecules or by genetically targeting cellular motor proteins (Maître et al., 2016). To change these in a temporally controlled manner, microfluidics or optogenetic approaches can be applied (Guglielmi et al., 2015; Valon et al., 2017). Small molecule inhibitors targeting the cytoskeleton or solutions with varied osmolarity could be applied in pulses or in spatial gradients using microfluidics to modulate mechanical forces and blastocoel formation.

Second, signaling pathways within the ICM and in-between embryonic and extraembryonic tissues have been indicated to guide the self-organization process. For instance, leukemia inhibitory factor (Lif) is expressed by TE cells and signals to the ICM (Nichols et al., 1996). Conversely, Bmp is expressed by ICM cells to control the surrounding extraembryonic cells (Nichols et al., 1996). Intercellular communication via fibroblast growth factor (FGF) controls the specification of Epi and PE cells in the ICM (Bessonnard et al., 2014). Such reciprocal communication could be manipulated by controlling the temporal addition of pathway modulators using microfluidics. To address the inter-cell-type interactions, it will be important to target a subset of cells, for instance using cell-specific activation of optogenetic tools (Repina et al., 2019; Toettcher et al., 2013). Alternatively, cells can be genetically manipulated; for instance, to be responsive to induction by tetracycline. If ELSs are derived from a combination of such cells with wild-type cells, some ICM cells could be induced to secrete FGF at defined time points.

### Engineering peri-implantation development

The blastocyst implants into the maternal endometrium, where it develops further by lineage specifications to form the three germ layers, a process called gastrulation. Besides receiving nutrients and oxygen from the mother, implantation of the blastocyst has two main consequences for the developing embryo. First, maternal tissue provides 3D support and could have instructive effects by mechanical signaling. Second, maternal factors induce implantation and might subsequently affect the self-organization process of the embryo.

To bridge the step from blastula to gastrula, implantation has been modeled *in vitro* using animal-derived 3D matrices such as collagen or Matrigel (Bedzhov and Zernicka-Goetz, 2014; Zheng et al., 2019). Models using human PSCs grown in a 3D matrix form both epiblast and amniotic ectoderm and initiate gastrulation (Shao et al., 2017; Zheng et al., 2019). Co-culture of mESC, TS, and extraembryonic endoderm stem (XEN) cells allows the formation of so-called ETX embryos, which display characteristics of the inner part of gastrulating mouse embryos, such as morphogenetic movements and gene expression, even in the absence of implantation (Figure 1B). However, gastrulation and the resulting structures are not complete (Shao et al., 2017; Sozen et al., 2018). Approaches based on 3D matrices could be optimized using chemically defined hydrogels with specific characteristics, such as modular stiffness, degradability, or composition of ECM ligands (Gjorevski et al., 2016). Matrices can be engineered to contain localized signaling centers or allow localized release of signaling molecules (Broguiere et al., 2020). Soon after implantation of blastoids *in utero*, development fails and blastoids are resorbed. FGF secretion from Epi cells is critical for remodeling the trophectoderm, allowing implantation (Nichols et al., 1998; Tanaka et al., 1998). Therefore, external localized activation of FGF signaling might support further development after implantation. Long-term organoid cultures of human endometrium have been established (Turco et al., 2017). Co-cultures of ELSs with endometrial organoids could serve as a cellular model of implantation and reveal reciprocal interactions between tissues.

### Engineering post-implantation development

During post-implantation development, the embryo undergoes gastrulation and substantial morphogenetic changes to establish a3D, elongating structure. This stage has been modeled in two dimensions by inducing the differentiation of hESCs on micropatterned circles, which results in the formation of radially symmetrical structures with concentric rings expressing markers of the three germ layers (Figure 1B) (Warmflash et al., 2014). Combined with the application of growth factor gradients using microfluidics, this system can model pattern formation through antagonistic morphogen gradients (Manfrin et al., 2019).

**Figure 3:**
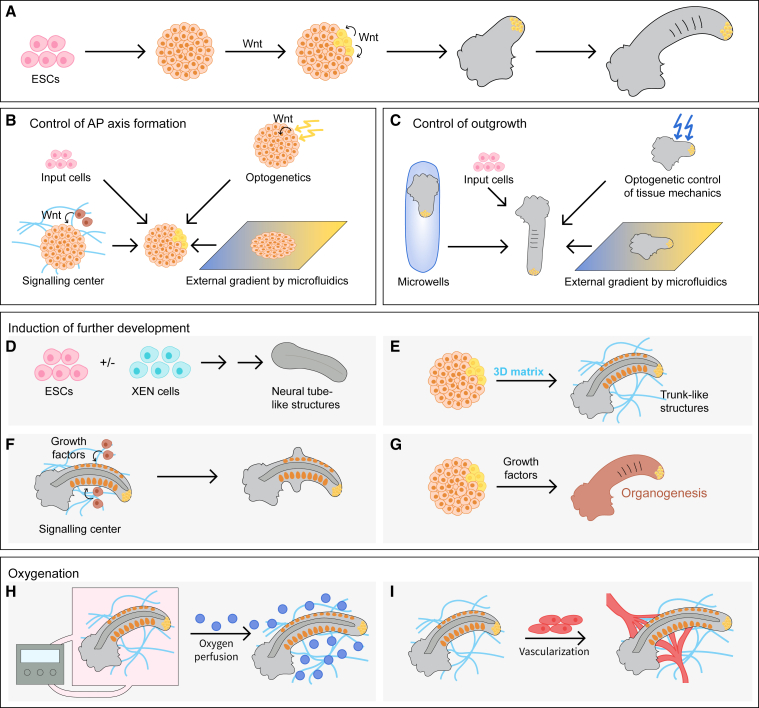
Bioengineering approaches in gastruloid research (A) Schematic description of gastruloid formation. ESCs are aggregated, Wnt is activated for 24 h, inducing AP axis and Wnt gradient. The posterior Wnt-high side (indicated in yellow) grows out. (B) AP axis formation is dependent on type and pluripotency of input cells and might be controlled externally by localized Wnt activation by Wnt-expressing cells, optogenetics, or microfluidics. (C) Amount and direction of gastruloid outgrowth can be guided by type and pluripotency state of input cells, growth within microwells, and might be controlled by optogenetics-based modulation of tissue mechanics or the continued application of an external Wnt gradient. (D–G) Modulation of the gastruloid protocol to allow further development. (D) Co-aggregating ESCs with XEN cells during the gastruloid protocol induces the formation of neural tube-like structures. (E) When gastruloids are cultured in a 3D matrix, neural tube and somite formation can be induced. (F) To induce, for instance, limb formation, growth factors could be provided locally, such as by printing growth factor-producing cells in the vicinity of gastruloids within ECM. (G) Modulation of gastruloid protocol by the addition of growth factors to induce organ formation. (H and I) To enable gastruloid growth for a longer period of time, oxygen availability might have to be optimized. This might be achieved by an external perfusion system (H) or by the co-culture with a perfused vascular system (I).

When grown in differentiation-permissive medium, PSC aggregates have the capacity to recapitulate key aspects of differentiation into cell types of all germ layers and morphogenesis. These so-called embryoid bodies self-organize a posterior pole with high Wnt activity in a Bmp-dependent manner (ten Berge et al., 2008). Upon transient external Wnt stimulation during this differentiation process, mESC aggregates self-organize into elongating structures with anteroposterior (AP) polarity termed gastruloids (Figures 1B and 3A) (Moris et al., 2020; van den Brink et al., 2014).

Despite this remarkable capability of gastruloids to self-organize, there is variability in cell composition, 3D organization, and overall shape. Bioengineering approaches can help to make gastruloids more reproducible by controlling external cues and boundary conditions. The number and pluripotency state of mESCs influence the efficiency of gastruloid formation and the composition of resulting cells (Cermola et al., 2021). After formation of a posterior pole, gastruloids elongate along the AP axis. To externally guide the location of outgrowth, the Wnt pulse required for gastruloid formation might be provided in a localized or graded manner, for instance, by localized Wnt secretion by signaling centers, patterning of growth factors (Batalov et al., 2021; Broguiere et al., 2020; Freeman et al., 2020), optogenetics (Repina et al., 2019), or microfluidics (Manfrin et al., 2019) (Figure 3B). The amount and direction of growth vary between gastruloids (van den Brink et al., 2020). Akin to engineering approaches in organoid research, gastruloid formation could be controlled by properly dimensioned microwells (Girgin and Lutolf, 2020; Hwang and Lee, 2011), optogenetic control of tissue mechanics (Guglielmi et al., 2015), external Wnt or FGF gradients, or polymeric microfilaments as scaffolds (Lancaster et al., 2017) (Figure 3C).

Even though gastruloids contain cell types of all germ layers, they lack the outer layer of surface ectoderm, which provides mechanical support in embryos and can be a source of growth factors. To establish this layer *in vitro*, cells might be co-cultured with surface ectoderm progenitor cells during gastruloid formation. Indeed, aggregating ESCs with XEN cells or TSCs can induce the formation of stratified neural tube-like structures or models of anterior development, respectively (Bérenger-Currias et al., 2020; Girgin et al., 2021) (Figure 3D). When gastruloids are grown in 3D matrices, formation of somite-like and neural tube-like structures is induced, implying an instructive effect of ECM on development (van den Brink et al., 2020; Veenvliet et al., 2020) (Figure 3E). A detailed analysis of required ECM components could guide the establishment of artificial matrices and make the protocol more reproducible.

In addition to providing mechanical cues, surface ectoderm provides signaling centers to organize development of the underlying tissue. For instance, the apical ectodermal ridge induces limb-bud formation by secreting FGFs (Sun et al., 2002). *In vitro*, localized sources of signaling molecules might be provided by artificial signaling centers in a 3D matrix (Broguiere et al., 2020; Freeman et al., 2020), morphogen-soaked beads, microfluidics (Manfrin et al., 2019), or the local activation by optogenetics (Toettcher et al., 2013). In this way, further development beyond the initial gastruloid might be induced, including the formation of limbs or organs (Figures 3F and 3G). Likewise, Wnt inhibition during gastruloid generation can induce anterior neural structure formation (Girgin and Lutolf, 2020). Besides morphogen gradients, dynamic signaling guides proper development. An optogenetic system, based on the light-inducible expression of oscillatory factors, has been applied to control neural differentiation (Imayoshi et al., 2013). Microfluidic systems enable the external control of endogenous signaling oscillations in mouse embryonic tissue (Sonnen et al., 2018). Such tools could help to modulate the timing and size of forming structures within gastruloids.

Finally, overall gastruloid development arrests after approximately 7 to 9 days of incubation, presumably due to an insufficiency in oxygen supply (Rossi et al., 2021). Embryos and ELSs are grown in roller cultures or while shaking to increase local oxygen concentration. To allow real-time imaging in stationary cultures, embryos are grown under hyperoxic conditions, under increased pressure, or using perfusion systems (Piliszek et al., 2011) (Figure 3H). In fact, a protocol combining static and roller culture of mouse embryos allows *ex utero* development from before gastrulation to late organogenesis (Aguilera-Castrejon et al., 2021). Even if oxygen concentration is externally controlled, diffusion of oxygen and nutrients in bigger structures is limited. By bioprinting or laser ablation of channels within 3D matrices, vascular systems can be generated (Brassard et al., 2021; Cochrane et al., 2019). Such engineering approaches or the self-organization of vasculature within gastruloids (Rossi et al., 2021) in conjunction with microfluidics might allow the establishment of perfused ELSs in the future (Grebenyuk and Ranga, 2019) (Figure 3I).

## Engineering organogenesis

Organoids recapitulate key aspects of development and morphology of the respective organ. However, they are taken out of the context of the organism, often only contain one tissue type, and are several orders of magnitude smaller than their *in vivo* counterparts. Here we consider approaches to trigger tissue construction across scales based on guidance principles that may better replicate native tissue-building processes during organogenesis.

Cells must coordinate their activities across large tissue fields via biochemical, electrical, and physical signals. Synthetic biology approaches have enabled engineers to create artificial morphogen sender/receiver cell relationships on the centimeter scale (Sekine et al., 2018; Toda et al., 2020). Integrating such circuits into models of organogenesis could better coordinate initial conditions for subsequent patterning, such as branching morphogenesis or generating repetitive tissues. Another application of long-range signaling in embryos is to enable communication between cell populations of developmentally distant origin. For example, in kidney branching morphogenesis, the ureteric epithelium derived from anterior intermediate mesoderm invades metanephric mesenchyme derived from posterior intermediate mesoderm. The timing at which these tissue layers interact may be impossible to mimic in organoid models emerging from a single pool of induced PSCs (iPSCs) (Taguchi et al., 2014). Bioengineering tools that can position cells at the appropriate stage in the appropriate spatial context and with co-patterning of instructive cues will be critical.

Besides morphogen signaling, electrical fields created through plasma membrane depolarization can be transmitted between cells via gap junctions or ion channels. Changes in membrane potential can then determine intracellular ion concentrations, affecting signaling networks associated with proliferation, apoptosis, or differentiation (Silver et al., 2020). Bioengineering tools can interact with and spatially pattern endogenous electric fields to shepherd collective cell motility in a process known as electrotaxis (Zajdel et al., 2020). This could create spatiotemporally programmed cell interfaces of precise shapes to trigger morphogenetic transitions.

**Figure 4:**
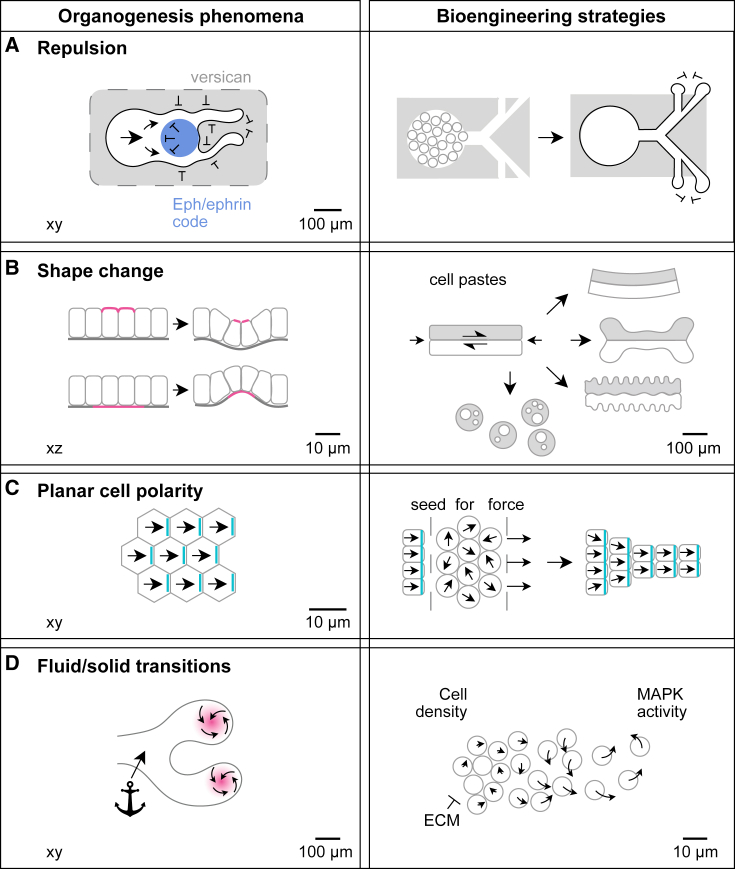
Bioengineering tools in organogenesis (A) Repulsion mechanisms operating at cell-cell, cell-ECM, and paracrine levels could be spatially patterned to shepherd changes in bioprinted tissue shape. (B) Guided shape change at cell paste interfaces could be generated through external and internally generated cell-cell or cell-ECM forces. (C) Planar cell (in-plane) polarity of cell collectives could be manipulated by spatial patterning of a pre-polarized seed, external force, or a biochemical gradient such as Wnt. (D) Gradients in cell density and mitogen signaling could stimulate formation of fluid-like and solid-like domains to guide dynamic remodeling of tissues.

Organogenesis relies upon cell integration of biochemical and physical cues from the microenvironment. Physical cues, including stiffness, viscoelasticity, and alignment of ECM components, can trigger collective cell migration and differentiation. Biochemical cues also mimic physical repulsion; for example, neural crest avoidance of cells expressing incompatible eph/ephrin “code” ligands or ECM components such as versican (Szabó et al., 2016). Spatially precise fabrication of such collective cell interfaces could guide tissue formation (Figure 4A). Not only do cells respond to external physical cues, they also create physical changes themselves that feedback on their own behavior (Hannezo and Heisenberg, 2019). New force-responsive materials could hijack such “call-and-response” interactions by tuning biomolecule release in response to local cell behaviors (Stejskalová et al., 2019).

Morphogenesis is crucial in development and organogenesis to form cavities (heart, lung), increase surface area (gut villi, brain cortex), or place stem cell niches in protected pockets (hair follicles, gut crypts). Shape change is driven by strain at interfaces caused by movement of cells relative to their surrounding matrix or tension imposed at the cell level. These strains can affect cellular differentiation by mechanotransduction (Mammoto et al., 2011). *In vitro* shape change can be engineered by mimicking boundary conditions that restrict tissue expansion to force interfacial buckling (Karzbrun et al., 2018), spatially patterning cell strains to create creases in ECM materials, or engineering the relative strengths of cell-matrix and cell-cell adhesion (Wang et al., 2020) (Figure 4B). In the future, it should be explored how inputs relate to shape change in, for example, bioprinted cell pastes (Lawlor et al., 2021). Future efforts should also address other interfacial strain mechanisms such as planar cell polarity, which sets up a planar reference frame in cell populations (Butler and Wallingford, 2017). The planar polarity axes could be biased by patterning pre-aligned material or cell “seeds” (Cohen et al., 2016) (Figure 4C) or by applying strain fields that prime cells for intercalation and oriented cell divisions (Aw et al., 2016).

Finally, the physical features of cells themselves account for a remarkable range of morphogenetic events. Manipulating these can affect organogenesis directly. For example, the expression levels and types of cell-cell adhesion molecules, such as cadherins, can yield predictable spatial sorting of cells (Steinberg, 2007; Wang et al., 2020). The density and motility of cells determine whether tissues are capable of being deformed by global forces or resist them by acting as “jammed” solids (Mongera et al., 2018). *In vitro*, one can manipulate the balance of “stabilizing” with “destabilizing” cell behaviors to guide organogenesis. For example, branching morphogenesis could be guided by spatially activating fluid-like tip cell collectives through optogenetic stimulation of MAPK signaling to self-renew and advance in an *in vitro* 3D environment (Figure 4D).

## Modeling organism-level development

**Figure 5:**
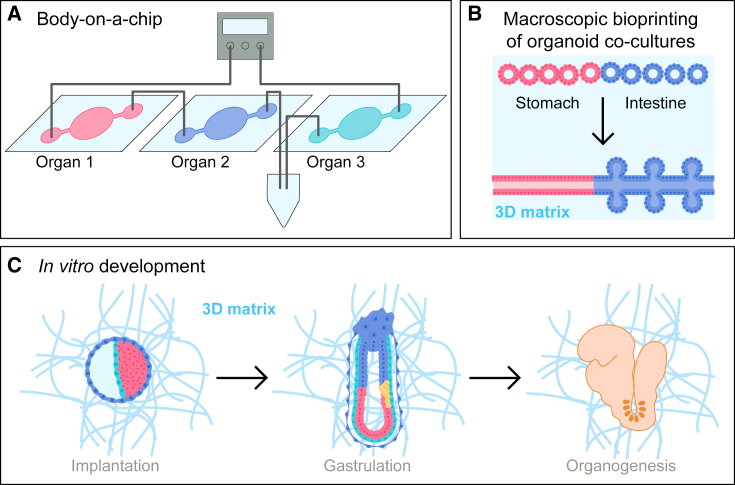
Toward *in vitro* modeling of organisms (A) Body-on-a-chip approaches aim at modeling the inter-connectivity between different organ types. (B) To build multi-organ structures representing the physiological context within the organism better, 3D bioprinting of different organoid types in close vicinity can be performed. (C) Guided self-organization of early embryos or ELS within *in vitro* implantation models is likely the best way to model the complex process of embryonic development *in vitro*.

With progressing organoid research, attempts to rebuild organisms or complex multi-organ units *in vitro* have advanced considerably. The basic principle is the co-culture of several functional parts in one reaction vessel, either in an open culture dish or within microfluidic chips encompassing multiple interconnected compartments (Figures 5A and 5B) (Zhang et al., 2009). The transition from organ-on-a-chip toward body-on-a-chip systems is exemplified by assembly of lung, liver, and heart cultures in a microfluidic setup to model metabolic reactions and drug toxicity (Skardal et al., 2017) (Figure 5A). Rather than connecting organs by microfluidic tubing, different organoid types can be bioprinted next to each other into a 3D matrix, allowing the self-organization of more complex organ model combinations, such as tubular structures of connected stomach and intestinal tissue (Brassard et al., 2021) (Figure 5B).

To date, these or similar approaches have been used successfully to model specific steps of development or parts of an organ or organism. Despite this progress in bioengineering, the self-organization capability of cells is still more competent in generating complex structures. Gastruloids can, for instance, form somites and neural tube (van den Brink et al., 2020; Veenvliet et al., 2020) or recapitulate early steps of heart formation (Rossi et al., 2021) (Figure 3). If one succeeded in generating a pre-implantation ELS that can be cultured in *in vitro* implantation models (Bedzhov and Zernicka-Goetz, 2014; Zheng et al., 2019), these structures might start organogenesis at the right time and the right place with proper interactions in between organs (Figure 5C).

## Discussion

Over the past decade, the development of stem cell-based *in vitro* models of embryonic development and adult tissues has revolutionized developmental biology. While some model systems are still in their infancy, others have entered the laboratory stage as standard model systems, such as gastruloids and organoids.

Several key challenges remain in the generation of stem cell-based *in vitro* models. One of the most pressing is to increase the reproducibility of *in vitro* cultures to be able to generate large numbers of uniform ELSs that do not vary within or between experiments or between research groups. Although *in utero* development is comparatively robust, ELSs are more diverse, even within a single experiment. One important variable here is the type and pluripotency state of input cells. Depending on the source, passage number, and culture condition, there will be subtle differences in cell state that directly affect the characteristics of forming cultures. Standardizing cell culture types and definition of protocols for testing the pluripotency state of input cells prior to ELS formation could minimize such variations.

With ever-improving *in vitro* systems, the use of research animals can be greatly reduced. Functional studies and high-throughput screens can be performed with stem cell-based models instead of embryos. However, currently available models do not fully recapitulate developing embryos. Even if ELSs efficiently model the process under investigation, confirmation in the embryo is essential. Depending on species, this confirmation should at least encompass expression analysis of key genes in fixed specimens or—if possible—functional perturbations in living embryos.

Here, we argue that advanced bioengineering approaches can contribute to the improvement of stem cell-based *in vitro* models of development. This could ultimately allow the development of organisms to be fully modeled *in vitro*. While this holds great promise for advancing our understanding of development and reducing the number of animals used in scientific research, this also brings ethical challenges (Rivron et al., 2018a). It must be clearly stated that ELSs are not synthetic embryos and can never be used in reproductive medicine. Besides this, depending on the type of ELS system and its resemblance to the actual embryo, their use in research needs to be monitored and controlled in the same way as animal models.

Following Richard Feynman's statement, combining bioengineering approaches with stem cell-based ELSs will bring us closer to understanding the full process of embryonic development: “What I cannot create I do not understand.”

## Declaration of interests

The authors declare no competing interests.
